# A social exclusion perspective on loneliness in older adults in the Nordic countries

**DOI:** 10.1007/s10433-022-00692-4

**Published:** 2022-03-29

**Authors:** Lena Dahlberg, Kevin J. McKee, Carin Lennartsson, Johan Rehnberg

**Affiliations:** 1 School of Health and Welfare, Dalarna University, 791 88 Falun, Sweden; 2 Aging Research Center, Karolinska Institutet and Stockholm University, Tomtebodavägen 18A, 171 65 Solna, Sweden; 3 Swedish Institute for Social Research, Stockholm University, 106 91 Stockholm, Sweden; 4 Department of Public Health Sciences, Stockholm University, 106 91 Stockholm, Sweden

**Keywords:** Social inclusion, Inequality, Social integration, Social isolation, Comparative

## Abstract

Several factors associated with loneliness are also considered indicators of social exclusion. While loneliness has been proposed as an outcome of social exclusion, there is limited empirical evidence of a link. This study examines the associations between social exclusion indicators and loneliness in older adults (60+ years) in four Nordic countries. Data from four waves of the European Social Survey were pooled, providing a total of 7755 respondents (Denmark *n* = 1647; Finland *n* = 2501, Norway *n* = 1540; Sweden *n* = 2067). Measures of loneliness, demographic characteristics, health, and eight indicators of social exclusion were selected from the survey for analysis. Country-specific and total sample hierarchical logistic regression models of loneliness were developed. Significant model improvement occurred for all models after social exclusion indicators were added to models containing only demographic and health variables. Country models explained between 15.1 (Finland) and 21.5% (Sweden) of the variance in loneliness. Lower frequency of social contacts and living alone compared to in a two-person household was associated with a higher probability of loneliness in all countries, while other indicators were associated with loneliness in specific countries: lower neighbourhood safety (Sweden and Denmark); income concern (Sweden and Finland); and no emotional support (Denmark, Finland, and Sweden). A robust relationship was apparent between indicators of social exclusion and loneliness with the direction of associations being highly consistent across countries, even if their strength and statistical significance varied. Social exclusion has considerable potential for understanding and addressing risk factors for loneliness.

**Supplementary Information:**

The online version contains supplementary material available at 10.1007/s10433-022-00692-4.

## Introduction

Loneliness has been defined as a perceived discrepancy between an individual’s desired and experienced social relations (Perlman and Peplau 1981). This subjective evaluation of one’s social relations concerns both their number and quality/intimacy (de Jong Gierveld 1998). Loneliness is associated with low well-being, poor physical and mental health, and mortality (e.g. Rico-Uribe et al. 2018; Solmi et al. 2020; Van As et al. 2021). Although an individual experience, societal perspectives can contribute to the understanding of loneliness (de Jong Gierveld and Tesch-Römer 2012). For example, some factors that increase the risk of loneliness in older adults operate primarily at the interpersonal or societal level, such as more limited social networks, lower levels of social activity, and less safe neighbourhoods (Dahlberg et al. 2022; Morgan et al. 2021). Some of these factors are also regarded as indicators of social exclusion, defined as a process whereby individuals are prevented from participating fully in activities regarded as standard for the society in which they live (Burchardt et al. 2002). Given this overlap of factors associated with loneliness and indicators of social exclusion, it is not surprising that a link between loneliness and social exclusion has been suggested with, for example, exclusion from social relations argued to increase the risk of loneliness (Burholt et al. 2020). However, little empirical research has been undertaken to examine this link. In this article, we explore the relationship between indicators of social exclusion and loneliness among older adults in the Nordic countries.

### Loneliness from a social exclusion perspective

Health and well-being are unequally distributed in society partly as a result of differential access to and possession of resources (Mackenbach 2019). Similarly, one key perspective on loneliness is that it is an outcome of limited access to resources that can help individuals to maintain activities that counteract loneliness (Tesch-Römer and Huxhold 2019). While research on inequalities primarily focuses on socio-economic resources, this resource perspective on loneliness is broader since it considers access to material and non-material resources, such as health, income, and socially responsive neighbourhoods, as influencing loneliness (Tesch-Römer and Huxhold 2019). There are similarities between the resource perspective on loneliness and how social exclusion is usually conceptualised, which is as a limited access to resources and activities across specific life domains (e.g. Dahlberg 2021; Silver 1994; Torres 2018).

Domains included in a conceptualisation of old-age exclusion proposed by Walsh et al. (2017) are: civic participation, social relations, neighbourhood, material resources, access to services, and sociocultural exclusion (Walsh et al. 2017; see also Van Regenmortel et al. 2016). Walsh and colleagues (2017) argue that exclusion has implications for both individuals, communities and societies at large. One plausible mechanism for the link between social exclusion and loneliness is that as an individual experiences greater exclusion and thus less access to a range of resources and activities across life domains, loneliness arises due to an inability to maintain such activities. In a recently proposed conceptual model, it has more specifically been argued that exclusion from social relations increases the risk of loneliness (Burholt et al. 2020).

From the limited empirical research to date that considers both social exclusion and loneliness, loneliness has been found to be an outcome of exclusion from social relations, from material resources and from the neighbourhood (Dahlberg and McKee 2014; Morgan et al. 2021; Myck et al. 2021), while societal-level indicators of social exclusion such as risk of poverty, material deprivation, community safety, and social connectedness are also associated with loneliness (Morgan et al. 2021). To our knowledge, there is no research exploring how other domains of exclusion, such as civic participation, are related to loneliness.

Additionally, many studies that do not explicitly use a social exclusion perspective have nevertheless provided evidence for associations between several indicators of social exclusion and loneliness. These indicators include widowhood, level of social contacts, income, financial concern, membership in organisations and neighbourhood activity, social engagement, community integration, and length of residence in a community (for a review on correlates and predictors of loneliness, see Cohen-Mansfield et al. 2016; for a systematic review of longitudinal risk factors of loneliness, see Dahlberg et al. 2022; for an umbrella review of factors associated with loneliness in the general population, see Solmi et al. 2020; see also de Koning et al. 2017; Power et al. 2019; Szabo et al. 2020).

### Social exclusion and loneliness in the Nordic countries

Given national/cultural variations in norms for social relations and access to resources, how social exclusion and loneliness both manifest and relate to each other is likely to differ between countries. Examining the pattern of associations between indicators of social exclusion and loneliness in different countries can therefore provide a valuable insight into which indicators are consistently associated with loneliness across countries, and which uniquely within countries. However, comparisons between highly dissimilar countries with, for example, different welfare systems, cultural norms, social structures, and disparities in wealth and health, are problematic as the many differences will obscure the relationship between social exclusion and loneliness. By contrast, comparisons between groups of countries with a shared heritage, similar cultures and norms, and close relations will provide less ‘background noise’ when examining the associations between social exclusion and loneliness (see Anckar 2008). In this respect, the Nordic countries provide an excellent setting for examining the potential for social exclusion as a framework for understanding loneliness in older adults.

Cross-country differences in loneliness have been discussed in relation to welfare state systems, where the Nordic welfare regime with an extensive welfare state is often held in high regard and proposed to be socially enabling, making older adults less dependent on individual resources (Nyqvist et al. 2019). While the Nordic countries collectively have a relatively low prevalence of loneliness (e.g. Fokkema et al. 2012; Hansen and Slagsvold 2015; Nyqvist et al. 2019; Swader 2019), treating these countries as one group hides inter-country variations. For example, a higher prevalence of loneliness has been observed in Finland and Sweden than in Denmark and Norway (Fokkema et al. 2012; see also Morgan et al. 2021; Sundström et al. 2009; Yang and Victor 2011). There are also welfare differences between the countries with, for example, greater social care coverage (combined residential care and home help) in Denmark and Norway than in Sweden and Finland (Rostgaard et al. 2022; Szebehely and Meagher 2018), and in income inequalities among older adults, with a higher at-risk-of-poverty rate among older adults (65+) in Sweden than other Nordic countries (Statistics Sweden 2017).

## Aim

The aim of this study is to explore the potential of a social exclusion framework for understanding loneliness by examining associations between indicators of social exclusion and loneliness among older adults in the Nordic countries. Our research questions are:

1. How much variance in older adults’ loneliness in the Nordic countries is explained by indicators of social exclusion?
2. To what extent does the level of variance in loneliness explained by social exclusion indicators vary across the Nordic countries?
3. Does the pattern of associations between indicators of social exclusion and loneliness vary across the Nordic countries?

## Methods

### Design and participants

This study is based on data from the European Social Survey (ESS). ESS data are collected via face-to-face interviews and with a cross-sectional sampling procedure designed to achieve nationally representative samples of individuals aged 15 years and over living in a private household, which might consist of, for example, parents and their children, students sharing accommodation, or an individual living alone, but exclude persons living in institutional facilities such as prisons, care homes, or residential mental health settings. A measure of loneliness, our dependent variable (DV), was included in ESS waves 3 (2006), 5 (2010), 6 (2012), and 7 (2014), and it was data from these four waves that we analysed. We restricted our analyses to people aged 60 years and older from Denmark, Finland, Norway, and Sweden. (Iceland was excluded as it participated in only one of the above survey waves.)

The prevalence of loneliness was relatively stable in each country across these waves (see Supplementary material). Therefore, after excluding respondents with internal missing values, we pooled data from these waves to generate one dataset with 1647 observations in Denmark, 2501 in Finland, 1540 in Norway, and 2067 in Sweden, *N* = 7755. Response rates across the four waves were only available for the total country samples: Denmark 49–55%; Finland 60–67%; Norway 54–66%; and Sweden 50–66%. ESS provides weights—based on age group, gender, education and region—that account for non-response biases (see Data analysis section).

### Materials

We selected three categories of variables from the ESS survey: 1) the DV, loneliness; 2) independent variables (IVs) that are regarded as indicators of social exclusion; and 3) IVs including demographic characteristics and health variables, selected both for describing the sample and due to their potential association with social exclusion and/or loneliness.

#### Dependent variable

*Loneliness* was measured by the question ‘how much of the time during the past week have you felt lonely’, with four response alternatives: none or almost none of the time; some of the time; most of the time; all or almost all the time. The frequencies for the higher response categories of the item were very low (see Supplementary material), and so the measure of loneliness was dichotomised into those who had not felt lonely at all (0), and those that had felt lonely some of the time or more during the last week (1).

#### Social exclusion indicators

Four domains of social exclusion were represented by the selected indicators: civic participation; social relations; material resources; and neighbourhood. All social exclusion indicators were coded in our analyses so that higher values indicated higher levels of exclusion.

*Civic exclusion* contained two indicators. The first indicator was a summary of five items on *political participation* during the last 12 months. The five items were yes/no questions on whether the respondent had: contacted a politician or government official; worn or displayed a campaign badge/sticker; signed a petition; taken part in a lawful public demonstration; or boycotted certain products. A yes response to one or more item was coded 0 for the indicator, and any other pattern of responses was coded 1. The second indicator was *voting behaviour*, measured as participation in the last national election with two responses (voted = 0; did not vote = 1).

*Exclusion from social relations* contained three indicators. *Social contacts* indicated how often the respondent met with friends, relatives, or colleagues with seven response options from every day (0) to never (7). *Emotional support* concerned whether the respondent had anyone with whom to discuss personal matters (yes = 0; no = 1). The third variable measured *household size*: single household; two-person household; and three plus-person household.

*Exclusion from material resources* contained two indicators. For *household income,* the population income distribution within each country was represented in deciles and each respondent coded on the basis of the location of their net income within that distribution (higher values indicated lower income). *Income concern* was measured by a question on whether the respondent felt that they were having difficulties or were not managing on their current income (no concerns = 0; concerns = 1).

Lastly, *neighbourhood exclusion* contained one indicator on neighbourhood safety, where the respondent rated her/his feeling of safety when walking alone in the local area after dark from very safe (0) to very unsafe (3).

#### Demographic and health variables

We included three demographic variables: *age* measured in years, *education* measured in years, and *gender* (male = 0, female = 1). Two variables were related to health status: *health-related limitations*, i.e. whether the respondent felt limited in his/her daily activities by any health-related problems from no (0) to yes a lot (2), and *self-rated health* from very good (0) to very bad (4).

### Data analysis

Hierarchical logistic regression was used to analyse the relationship between social exclusion and loneliness in the Nordic countries. Hierarchical regression is a method for evaluating the ‘added value’ in terms of the explained variance in a DV due to a set of IVs that are entered into a regression model after other IVs, thus statistically controlling the effect of the first set of IVs. We estimated the variance in loneliness explained by all domains of social exclusion by running models in which loneliness was regressed on 1) only demographic and health variables and 2) all variables including social exclusion indicators. The increment in variance in loneliness explained by model 2 relative to model 1 (model fit improvement) represents the additional variance in loneliness accounted for by social exclusion.

We evaluated the fit of our models in two ways. First, we report two pseudo* R*^2^ statistics: Nagelkerke’s and Efron’s. These are equivalents to the *R*^2^ statistic used in linear regression that represents explained variance in a DV. The two pseudo *R*^2^ statistics are calculated differently, and so we include both. Second, we report the Akaike information criterion (AIC), which quantifies the relative performance of a statistical model (Akaike 1973). Model performance is assessed by comparing AIC values between models, where lower values indicate better performance. A reduction of 10 in the AIC value when comparing models has been suggested as sufficient for accepting the model with the lower AIC value as superior (Burnham and Anderson 2004).

We first performed analyses on the total sample, thus addressing our first research question. To assess potential variation across the Nordic countries, the analyses were then stratified by country, thus addressing our second and third research questions. The presence of multicollinearity was checked with the variance inflation factor (VIF), which indicated low correlation between variables (highest value 1.91). We estimated average marginal effects (AMEs) for all social exclusion variables in the final models. AMEs estimated from a logistic regression model are interpreted as the probability of the average person in the data experiencing the outcome. We applied the ESS-provided weights to all regression analyses and when reporting the level of loneliness. When describing the independent variables in the sample (Table 1), we did not apply weights, as the intention was not to make inferences to the populations from where the samples were drawn but to describe sample characteristics.

**Table 1:** Descriptive statistics for all study variables by country

	Denmark	Finland	Norway	Sweden
*N*	1647	2501	1540	2067
	%			
Loneliness^1^	16.6	23.3	18.8	23.6
	Mean (SD, range)			
Age (years)	69.7 (7.5, 60–95)	70 (7.7, 60–97)	69.9 (7.8, 60–104)	70.2 (7.6, 60–114)
Education (years)	12.0 (5.1)	10.8 (4.2)	12.0 (4.2)	11.4 (4)
Household income decile^2^	6.4 (2.6)	6.2 (2.4)	6.6 (2.6)	5.6 (2.8)
	%	%	%	%
*Gender*				
Male	52.0	46.9	51.3	51.0
Female	48.0	53.1	48.7	49.0
*Health-related limitations*				
No	67.6	54.8	64.9	60.3
Yes, to some extent	25.1	33.7	26.8	30.4
Yes, a lot	7.3	11.5	8.2	9.2
*Self-rated health*				
Very good	31.8	8.4	21.1	23.3
Good	34.4	36.4	42.9	44.1
Fair	26.2	46.9	28.8	26.9
Bad	6.3	7.3	6.2	4.5
Very bad	1.3	1.0	1.0	1.3
*Political participation*				
No	54.6	52.4	46.8	35.6
Yes	45.4	47.6	53.2	64.4
*Voting behaviour*				
Voted	95.2	88.8	90.3	93.7
Did not vote	3.6	11.1	7.9	5.1
*Social contacts*				
Everyday	11.9	12.2	12.1	13.7
Several times per week	36.6	24.6	37.7	31.2
Once a week	20.3	22.1	17.4	19.4
Several times a month	20.6	22.2	21.9	24.1
Once a month	7.4	11.3	6.9	7.3
Less than once a month	2.9	7.6	3.8	3.9
Never	0.3	0.2	0.3	0.4
*Emotional support*				
Yes	90.5	91.6	90.9	92.5
No	9.5	8.4	9.1	7.5
Household size				
Single household	28.8	34.4	28.9	31.4
Two-person household	67.5	61.3	66.3	64.1
Three plus-person household	3.8	4.3	4.8	4.5
*Income concern*				
No concerns	96.1	89.7	95.3	91.8
Concerns	3.9	10.3	4.7	8.2
*Neighbourhood safety*				
Very safe	48.1	33.5	44.7	39.2
Safe	39.2	53.9	42.7	43.1
Unsafe	9.2	10.3	10.4	13.5
Very unsafe	3.5	2.3	2.2	4.2

^1^Weighted frequency

^2^1 indicates the highest 10% incomes; 10 indicates the lowest 10% incomes

## Results

### Descriptive analyses

Table 1 presents descriptive analyses for all study variables by country. Loneliness was higher in Sweden (23.6%) and Finland (23.3%), somewhat lower in Norway (18.8%) and lowest in Denmark (16.6%). Regarding demographic and health characteristics, mean age was around 70 in all four countries, while the length of education ranged from 10.8 to 12.0 years. There were slightly more male than female respondents in Denmark (52.0%), Norway (51.3%), and Sweden (51.0%), and more female respondents in Finland (53.1%). Between 54.8 and 67.6% of the sample had no health-related limitations. Self-rated health was good or very good for around two thirds of each country sample, except in Finland where this proportion was 44.8%.

Regarding the social exclusion indicators, political participation ranged from 45.4% in Denmark to 64.4% in Sweden. The proportion of respondents voting in the previous national election varied from 88.8% in Finland to 95.2% in Denmark. Around two-thirds of each country sample had social contacts at least weekly, although this proportion was slightly less than 60% in Finland. Most respondents had emotional support, ranging from 90.5 (Denmark) to 92.5% (Sweden). Approximately a third of respondents lived in single households (from 28.8% in Denmark to 34.4% in Finland), with a majority living in two-person households (from 61.3% in Finland to 67.5% in Denmark). While most respondents had no income concerns, such concerns were more common in Finland (10.3%) and Sweden (8.2%) than in Norway (4.7%) and Denmark (3.9%). The proportion of respondents who felt unsafe or very unsafe in their neighbourhood was almost identical in Denmark, Finland, and Norway (12.6–12.7%), but higher in Sweden (17.7%).

### Multivariable analyses of Nordic sample

Table 2 presents the results of the fully adjusted (final) logistic regression model for the total sample for loneliness regressed on demographic, health, and social exclusion variables. The model fit indices showed large improvements from model 1 (demographic and health variables only entered) to model 2 (all IVs entered). The Nagelkerke *R*^2^ value increased from 0.058 in model 1 to 0.186 in model 2, an increase in explained variance of 12.8%. Similarly, Efron’s *R*^2^ increased from 0.056 to 0.151, and increase in explained variance of 9.5%. The change in the AIC value was − 671, indicating substantial and significant improvement in model fit due to the addition of social exclusion indicators.

**Table 2:** Logistic regression of loneliness on demographic, health, and social exclusion variables in total sample, fully adjusted (final) model (*N* = 7755)

Variables	OR	Lower 95% CI	Upper 95% CI	*p*
Intercept	0.007	0.003	0.014	0.000
*Demographic and health variables*				
Age (years)	1.017	1.009	1.025	0.000
Education (years)	1.002	0.985	1.018	0.823
Gender (ref = male)	1.021	0.891	1.169	0.765
Health-related limitations (ref = no)				
Yes, to some extent	1.349	1.163	1.564	0.000
Yes, a lot	1.478	1.177	1.853	0.001
Self-rated health (higher values, worse health)	1.227	1.128	1.336	0.000
*Social exclusion variables*				
No political participation (ref = political participation)	0.938	0.825	1.066	0.324
Did not vote (ref = voted)	1.268	1.033	1.551	0.022
Infrequent social contacts (higher values, less contacts)	1.185	1.132	1.240	0.000
No emotional support (ref = emotional contact)	1.514	1.244	1.839	0.000
Household size (ref = two-person household)				
Three plus-person household	1.494	1.077	2.038	0.013
Single household	4.581	3.920	5.360	0.000
Lower household income (higher values, lower income)	0.974	0.942	1.007	0.121
Income concern (ref = no concerns)	1.648	1.338	2.028	0.000
Lower neighbourhood safety (higher values, lower safety)	1.145	1.054	1.244	0.001

Model fit	Model 1: demographic and health	Model 2: final model	Change
Nagelkerke *R*^2^	0.058	0.186	0.128
Efron’s *R*^2^	0.056	0.151	0.095
AIC	7636	6965	− 671

*OR* odds ratio; *CI* confidence interval; *ref* reference category

Of the demographic and health variables, greater age was associated with increased odds of loneliness, as was poorer self-rated health and having moderate or high levels of health limitations relative to having no health limitations.

Six out of the eight social exclusion indicators were significantly associated with loneliness. Not having voted in the last national election, having less frequent social contacts, having no emotional support, having income concerns and lower neighbourhood safety were all associated with an increased the risk of loneliness. There was a u-shaped association between household size and loneliness: compared with respondents living in two-person households, both respondents living in a three plus-person household and those living in a single household had an increased risk of loneliness.

### Multivariable analyses stratified by country

Tables 3 and 4 present the results of the fully adjusted (final) logistic regression models for loneliness regressed on demographic, health, and social exclusion variables by country.

**Table 3:** Logistic regression of loneliness on demographic, health, and social exclusion variables in Danish (*N* = 1647) and Finnish (*N* = 2501) samples, fully adjusted (final) models

	Denmark				Finland			
Variables	OR	Lower 95% CI	Upper 95% CI	*p*	OR	Lower 95% CI	Upper 95% CI	*p*
Intercept	0.004	0.001	0.022	0.000	0.011	0.003	0.040	0.000
*Demographic and health variables*								
Age (years)	1.007	0.987	1.027	0.500	1.015	1.001	1.030	0.042
Education (years)	0.999	0.966	1.033	0.938	1.010	0.979	1.042	0.530
Gender (ref = male)	1.065	0.773	1.468	0.702	0.979	0.771	1.243	0.862
Health-related limitations (ref = no)								
Yes, to some extent	1.122	0.772	1.623	0.543	1.582	1.230	2.036	0.000
Yes, a lot	1.510	0.863	2.620	0.145	1.706	1.168	2.481	0.005
Self-rated health (higher values, worse health)	1.333	1.107	1.608	0.002	1.135	0.961	1.341	0.138
*Social exclusion variables*								
No political participation (ref = political participation)	1.007	0.736	1.382	0.964	1.138	0.911	1.422	0.255
Did not vote (ref = voted)	1.389	0.793	2.372	0.238	1.145	0.827	1.572	0.409
Infrequent social contacts (higher values, less contacts)	1.320	1.179	1.479	0.000	1.151	1.067	1.241	0.000
No emotional support (ref = emotional support)	1.747	1.142	2.640	0.009	1.615	1.129	2.295	0.008
Household size (ref = two-person household)								
Three plus-person household	2.021	0.881	4.248	0.077	1.669	0.962	2.783	0.057
Single household	3.576	2.484	5.179	0.000	4.384	3.276	5.896	0.000
Lower household income (higher values, lower income)	1.013	0.932	1.103	0.763	0.978	0.914	1.047	0.527
Income concern (ref = no concerns)	1.691	0.907	3.070	0.090	1.488	1.072	2.058	0.017
Lower neighbourhood safety (higher values, lower safety)	1.209	1.002	1.456	0.047	0.998	0.856	1.163	0.980

Model fit	Model 1: demographic and health	Model 2: final model	Change	Model 1: demographic and health	Model 2: final model	Change
Nagelkerke *R*^2^	0.091	0.190	0.099	0.057	0.174	0.118
Efron’s *R*^2^	0.060	0.139	0.079	0.042	0.127	0.086
AIC	1312	1229	− 83	2630	2437	− 193

*OR* odds ratio; *CI* confidence interval; *ref* reference category

**Table 4:** Logistic regression of loneliness on demographic, health, and social exclusion variables in Norwegian (*N* = 1540) and Swedish (*N* = 2067) samples, fully adjusted (final) models

	Norway				Sweden			
Variables	OR	Lower 95% CI	Upper 95% CI	*p*	OR	Lower 95% CI	Upper 95% CI	*p*
Intercept	0.009	0.002	0.045	0.000	0.003	0.001	0.013	0.000
*Demographic and health variables*								
Age (years)	1.032	1.013	1.051	0.001	1.017	1.001	1.033	0.041
Education (years)	0.982	0.944	1.022	0.371	1.022	0.988	1.057	0.200
Gender (ref = male)	0.699	0.506	0.961	0.028	1.327	1.028	1.712	0.030
Health-related limitations (ref = no)								
Yes, to some extent	1.153	0.815	1.622	0.418	1.289	0.973	1.704	0.076
Yes, a lot	1.557	0.919	2.615	0.097	1.171	0.746	1.827	0.489
Self-rated health (higher values, worse health)	1.337	1.104	1.621	0.003	1.191	1.013	1.399	0.034
*Social exclusion variables*								
No political participation (ref = political participation)	0.757	0.561	1.018	0.067	0.905	0.704	1.159	0.430
Did not vote (ref = voted)	1.524	0.993	2.310	0.050	1.141	0.727	1.771	0.561
Infrequent social contacts (higher values, less contacts)	1.146	1.031	1.273	0.011	1.168	1.068	1.277	0.001
No emotional support (ref = emotional support)	1.104	0.716	1.681	0.649	1.731	1.157	2.575	0.007
Household size (ref = two-person household)								
Three plus-person household	0.953	0.419	1.941	0.900	1.570	0.820	2.821	0.150
Single household	5.023	3.569	7.110	0.000	5.166	3.847	6.972	0.000
Lower household income (higher values, lower income)	0.967	0.900	1.041	0.370	1.004	0.945	1.067	0.894
Income concern (ref = no concerns)	1.597	0.911	2.778	0.099	1.545	1.051	2.273	0.027
Lower neighbourhood safety (higher values, lower safety)	1.083	0.885	1.322	0.438	1.276	1.101	1.480	0.001

Model fit	Model 1: Demographic and health	Model 2: Final model	Change	Model 1: Demographic and health	Model 2: Final model	Change
Nagelkerke *R*^2^	0.034	0.168	0.134	0.059	0.232	0.173
Efron’s *R*^2^	0.072	0.171	0.099	0.066	0.197	0.131
AIC	1477	1358	− 119	2195	1949	− 246

*OR* odds ratio; *CI* confidence interval; and *ref* reference category

Comparing the final models, most variance in loneliness was explained in the Sweden sample (averaging the two pseudo *R*^2^ estimates, 21.5% variance) and least explained in the Finland sample (15.1%). The model fit indices in all countries showed substantial and significant improvements between model 1 and model 2, with the largest improvement in explained variance in the Swedish sample (average pseudo *R*^2^ increment, 15.1%), followed by Norway (11.7%), Finland (10.2%), and finally Denmark (8.9%). The pattern of model improvements indicated in the AIC estimates mostly reflected those of the pseudo *R*^2^ estimates, with the largest reduction between model 1 and model 2 in Sweden (− 246), followed by Finland (− 193), Norway (− 119), and lastly Denmark (− 83). Overall, these results indicate that adding social exclusion indicators to a model of loneliness significantly improved that model in all four Nordic countries, with both the largest improvement in explained variance in loneliness and the largest amount of total variance explained in loneliness in the Sweden sample.

**Fig. 1:**
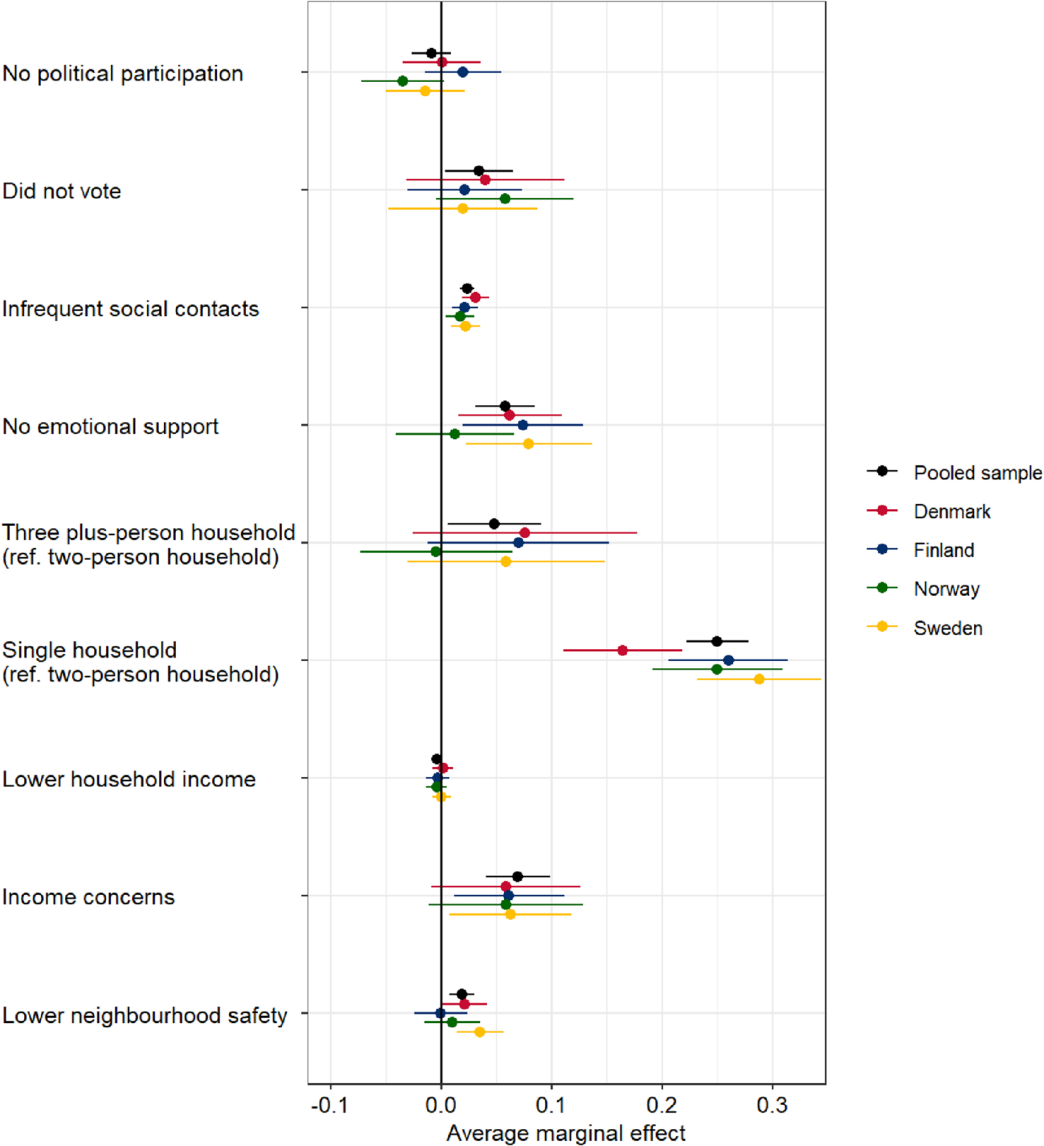
Average marginal effects on loneliness for all social exclusion variables by country and for total sample, estimated from Tables 2, 3, and 4

The AMEs for the social exclusion indicators from the fully adjusted logistic models in Tables 2, 3, and 4 are presented in Fig. 1 stratified by country and for the total sample. The figure displays the point estimate and 95% confidence intervals for the effect between each indicator and loneliness, with no significant association indicated if the confidence intervals cross 0, while estimates above 0 indicate increased risk of loneliness and estimates below 0 indicate decreased risk of loneliness. Below, we only consider those indicators that demonstrated a significant association with loneliness.

In the total sample, respondents who did not vote in the last election had a 3.46% point higher probability of loneliness compared to those that did vote. This association was not significant in the country analyses, although the associations were in the same direction and with similar strength in all countries.

Having less frequent social contacts was associated with increased loneliness in both total and country samples. In the total sample, for each unit decrease in the frequency of contact the probability of loneliness increased by 2.36% points.

No emotional support was associated with a higher probability of loneliness in all countries except Norway, with the largest effect found in Sweden. In the total sample, a respondent with no emotional support had a 5.79% point increase in the probability of loneliness compared to one with emotional support.

A u-shaped association between household size and loneliness was observed in the total sample. Respondents living in a three plus-person household had a 4.79% point higher probability of loneliness compared to those living in a two-person household, while respondents living in a single household had a 25.0% point higher probability of loneliness compared to those living in a two-person household. In Sweden, Finland and Denmark, the point estimates for living in a three plus-person household indicated a positive, but non-significant association with loneliness, while in Norway the point estimate was close to zero. The association between living in a single household and loneliness was significant and large also in the separate country analyses, if comparatively weaker in Denmark.

Income concern was significantly associated with a higher probability of loneliness in the total sample and in the Sweden and Finland samples, but not in the Denmark and Norway samples. In the total sample, respondents with income concerns had a 6.97% point higher probability of loneliness compared to those without income concerns.

Lastly, lower levels of perceived safety in the neighbourhood were significantly associated with an increased risk of being lonely in the total sample and in Sweden and Denmark, but not in Norway and Finland. In the total sample, a unit decrease in a respondent’s reported neighbourhood safety was associated with a 1.89% point higher probability of loneliness.

## Discussion

This study aimed to explore the potential of a social exclusion perspective for understanding loneliness by examining associations between indicators of social exclusion and loneliness among older adults in the Nordic countries. We posed three research questions. First, how much variance in older adults’ loneliness in the Nordic countries is explained by indicators of social exclusion? In the model which combined the samples for the four Nordic countries, we found that when social exclusion indicators were added to a model containing demographic and health variables, there was a significant improvement in the model. Taking the average of the two pseudo *R*^2^ statistics, the variance explained in loneliness increased by 11.2%. Second, to what extent does the level of variance in loneliness explained by social exclusion indicators vary across the Nordic countries? The variance explained in loneliness by the models for Denmark, Finland, and Norway was relatively similar (averaging the two pseudo *R*^2^ estimates, 16.5, 15.1 and 17.0%, respectively), with the variance explained in the Sweden sample somewhat higher (21.5%). The Sweden sample also demonstrated the greatest influence of social exclusion indicators, with the largest increment in explained variance (15.1%) of the country samples when social exclusion variables were added to models with demographic and health variables only. By comparison, the increment in variance in Norway was 11.7%, Finland 10.2%, and Denmark 8.9%. Third, does the pattern of associations between social exclusion indicators and loneliness vary across the Nordic countries? Our results indicate that less frequent social contacts and living in a single household compared to a two-person household were associated with loneliness in all countries. Having no emotional support was associated with loneliness in all countries except Norway; lower neighbourhood safety was associated with loneliness in Sweden and Denmark only; and having income concerns was only associated with loneliness in Sweden and Finland.

### Social exclusion and loneliness

Many diverse factors related to different life domains have been shown to be associated with loneliness in old age (Cohen-Mansfield et al. 2016; Dahlberg et al. 2022). In this study, we proposed that social exclusion has value as a perspective on loneliness. While a large number or risk factors for loneliness have been covered in previous research, a social exclusion perspective offers a framework for the selection, organisation, and interpretation of risk factors, i.e. provides a coherent narrative for understanding how various resources across different life domains are related to loneliness. Our reasoning was based on the suggested conceptual (Burholt et al. 2020) and demonstrated empirical (Morgan et al. 2021; Myck et al. 2021) connection between social exclusion and loneliness. This reasoning is also supported by studies that have examined social exclusion’s link with indicators of quality of life (Dahlberg and McKee 2018; Scharf et al. 2005). For example, a recent study found that while social exclusion was lower in Nordic countries than in Western, Central, and Eastern and Southern Europe, subjective well-being (measured as life satisfaction, happiness, and general health) was higher (Lee 2020). It could be argued that an inequalities perspective on loneliness offers a similar value to that provided by a social exclusion perspective. However, the inequalities perspective tends to focus on socio-economic and material resources only, whereas social exclusion offers a broader perspective that considers access to both material and non-material resources across several life domains, which resonates strongly with the resource perspective on loneliness (Tesch-Römer and Huxhold 2019).

When considering the results of our analysis of the total sample, we find support for the argument that social exclusion and loneliness are connected. Out of eight social exclusion indicators, six were associated with loneliness, and the addition of the social exclusion indicators produced significant increment in the variance in loneliness explained by the model. Furthermore, all four social exclusion domains included in our study were represented by these six indicators, suggesting that the breadth of the social exclusion perspective in encompassing a range of life domains has particular relevance for understanding loneliness.

When analysing the relationship between variables, it is always wise to take account of any clustering in the data, as relationships obtained when analysed in pooled data can fail to materialise or even manifest in the opposite direction when considered within clusters (cf. the ecological fallacy and Simpson’s paradox). Our analyses demonstrate a considerable degree of consistency in this respect, with the associations between social exclusion indicators and loneliness in the combined and country-level data in most instances having the same direction. However, and as would be expected, the significance and strength of the associations obtained between the social exclusion indicators and loneliness varied across the four countries.

Household size and social contacts were the two social exclusion indicators consistently significantly associated with loneliness in all four countries, where the strongest association with loneliness was for respondents living in single compared to two-person households. There is strong evidence in previous research that living alone increases the risk of loneliness (Cohen-Mansfield et al. 2016; Dahlberg et al. 2022). This group includes people who have never married, are divorced/separated, or widowed. Research focusing on marital status rather than cohabitation has similarly found an association between those who are single and loneliness (Cohen-Mansfield et al. 2016; Dahlberg et al. 2022). Our study also identified an increased risk of loneliness in those living in a three plus-person household when compared to a two-person household, but this association was only significant in the total sample and the effect size was considerably less than that for living in a single household. The majority of those living in a two-person household will be one member of a couple, while three plus-person households will vary in their composition and will arise due to a number of reasons, e.g. older parents living with their adult children, and ethnic and cultural norms for family size and extended families within a household. There is thus not a linear relationship between the number of people in the immediate living environment and loneliness. Rather it is the composition of the household that relates to loneliness, such that living with one other person provides some protection against loneliness when compared to living alone or in a larger household.

While having less frequent social contacts was consistently associated with loneliness in all four countries, the effect sizes were relatively small compared to those for living alone. Similarly, the effect sizes for the total and country associations between having no emotional support and loneliness were larger than those for frequency of social contacts, albeit that emotional support was not significantly related to loneliness in Norway. These findings correspond to the argument that the quality of social relations is more central for loneliness than quantity or frequency of social contacts (Cohen-Mansfield et al. 2016; Warner and Adams 2016), such that, for example, infrequent but emotionally supportive contacts provide greater protection against loneliness than frequent but emotionally unsatisfying contacts. However, when comparing effect sizes for association, the scale on which an IV is measured should be taken into consideration, as the effect size for a unit change in an IV measured over a range of values will likely be lower than that of an IV that has only two values. Both emotional support and household size were dichotomous IVs (the latter as a result of dummy coding), whereas frequency of social contacts was measured on a six-point scale.

The two indicators of exclusion from civic engagement contributed relatively little to the explanation of loneliness. Political participation was not associated with loneliness, while not voting in the previous election was associated with loneliness only in the total sample, albeit the associations for not voting with loneliness were in a consistent direction in each of the four countries, and their strengths similar. While it is difficult to construct an argument for not voting to be causally linked to loneliness, in the Nordic countries where voting is strongly normative, the act of not voting suggests a high level of civic disengagement. It is this experience of disengagement from civic life that might more reasonably be seen as contributing to a feeling of loneliness. Similar to exclusion from civic engagement, one of the two indicators of exclusion from material resources—household income—was not associated with loneliness. However, the other indicator—having income concerns—was significantly associated with loneliness in the total sample and in Finland and Sweden, but not in Denmark or Norway (cf. Cohen-Mansfield et al. 2009). Given that the effect sizes for the association between income concerns and loneliness were similar for the total sample and across the four countries, the non-significant associations for Denmark and Norway may as much reflect their smaller sample sizes relative to Finland and Sweden as they do meaningful cultural differences. These findings for the two indicators of exclusion from material resources suggest that loneliness is less related to absolute material wealth than to the perception of inadequate resources. This notion finds support in the health inequality literature where it has been proposed that that psychosocial pathways—based on, for example, stress caused by social comparisons and feelings of psychosocial disadvantage—partly explain the association between income and health (see Marmot 2005; Rehnberg 2019). The effects of these mechanisms have been proposed to be exacerbated when income inequalities increase (Pickett and Wilkinson 2015), which has been the case in the Nordic countries (OECD 2011). It is also important to consider that household income may not be a sufficient measure of the material standard of living, and thus, the effect of material wealth on loneliness may be underestimated in our analyses.

As with income concerns, neighbourhood safety was significantly associated with loneliness in the total sample and in only two countries. Although the effect sizes for the associations for neighbourhood safety across the individual countries did not vary greatly, those for Denmark and Sweden were distinctly larger than in Finland and Norway. Thus, neighbourhood safety appears to be the exclusion indicator that varies most across the countries in terms of its importance for loneliness. Compared to Norway and Finland, Sweden and Denmark have a larger proportion of their populations living in urban environments (CIA 2021), which may have implications for how neighbourhood safety and loneliness is experienced. One study found that neighbourhood safety had an influence on both neighbourhood attachment and satisfaction with social network, both of which were in turn associated with loneliness (Kemperman et al. 2019), while other studies have found loneliness to be associated with area deprivation (Victor and Pikhartova 2020) and low perceived community integration (Dahlberg and McKee 2014). Otherwise, little quantitative research has explored the role of neighbourhood in loneliness to date.

## Strengths and limitations

Given that there has been relatively little research on the relationship between social exclusion and loneliness, we would argue that this study is an original and valuable contribution to evaluating the potential of a social exclusion perspective for understanding loneliness. Analysing the data on social exclusion and loneliness from four different but culturally similar countries generates considerable confidence in the robustness of the observed associations due to the opportunity to compare the models of loneliness for the substantial and representative ESS samples from each country and for the total sample.

Data were pooled from four ESS data collection waves (2006–2014) that contained a measure of loneliness. The number of observations per wave was in many instances less than 500 persons, and those persons that experienced feelings of loneliness were rather few. A logistic regression model with many IVs and a DV with few observations in the rarer of its two categories is susceptible to small-sample bias, and pooling the data from different waves allowed us to circumvent this issue. While this procedure may have masked changes in the associations between indicators of social exclusion and loneliness over time, the prevalence of loneliness did not change substantially across waves in the four countries and additional analyses (not presented) performed separately for each wave showed no systematic deviations in findings from those obtained in the pooled data. As such, we believe that the pooling of the data across the four ESS waves to increase sample size for our analyses was warranted. Even with the pooling of data, the representation of important subgroups in our sample, such as ethnic minorities, was insufficient to allow for further analysis.

In this article, we propose a mechanism whereby an individual who experiences social exclusion, and thus less access to a range of resources and activities across different life domains, is at higher risk of loneliness. In this, we do not argue that each indicator of exclusion is itself a causal determinant of loneliness, but that each indicator of exclusion contributes to an experience of exclusion that increases the risk of loneliness. Clearly, a limitation of our study is that a cross-sectional design cannot confirm a causal hypothesis, and as such our findings are open to the interpretation that loneliness influences social exclusion rather than vice versa. Certainly, one could posit a ‘feedback loop’ in which exclusion elicits loneliness, which in turn exacerbates the experience of exclusion and may indeed create the conditions for an objective worsening of exclusion. While entertaining such a model, we do not see loneliness as a primary driver of exclusion.

A further limitation of our study is that it is based on secondary analyses of data from a study that was not designed to examine social exclusion. This restricted the selection of variables that could be justified as valid indicators of social exclusion and the range of domains to be included in the analyses. Furthermore, some of the variables selected as indicators were of limited sensitivity. For example, only one variable was suitable for inclusion in the analyses as an indicator of neighbourhood exclusion, while both indicators of exclusion from civic engagement were dichotomous.

There were also relatively skewed response distributions on some indicators, which will have reduced the power of our analyses to detect reliable effects for associations between such indicators and loneliness. As there were very few responses in the higher categories of the loneliness measure, we dichotomised the measure. While dichotomising a scale is never desirable, regressing a large number of variables onto a small number of observations can bias the parameter estimates in the model. In such circumstances, creating a cut-off to separate those respondents who to any degree experienced loneliness during the very limited timeframe of the previous week (feeling lonely some, most, or all of the time) from those who did not (feeling lonely none or almost none of the time) was the optimal solution.

Finally, the lower age for inclusion in the study sample was 60 years, which is relatively young to represent an older adult population, and the same findings may not have materialised if the sample had been restricted to an older age group.

## Conclusions

While a link between social exclusion and loneliness has been posited previously, there have been few studies that have tested the empirical basis of the link. The Nordic countries have similar cultures and close relationships, but also inter-country variations in social exclusion and loneliness, thus providing an excellent canvas on which to explore the connection between the two concepts. Indicators of social exclusion explained significant variance in loneliness in all four Nordic countries studied, with the indicators improving models that contained demographic and health variables. While the direction of the associations was highly consistent across countries, their strength and statistical significance varied. These findings suggest a robust relationship between social exclusion and loneliness, while also providing a corrective to the assumption that one should expect to explain loneliness to the same level, and via the same social exclusion factors, in even culturally and normatively similar countries. While confirming the relevance of social relations to loneliness, the findings also point towards the importance of going beyond a narrow focus on, for example, socio-economic inequalities when examining and in practice addressing factors associated with loneliness. Future research could extend our understanding of the value of social exclusion as an explanatory framework for loneliness by exploring the relationship between loneliness and other indicators of social exclusion, representing other life domains, in a more diverse range of countries. Given the argument that exclusion from one domain increases the risk of exclusion from other domains (e.g. Levitas et al. 2007; Walsh et al. 2017), a social exclusion perspective could form the basis for research on the combined effect of exclusion from different domains on loneliness. Finally, research using longitudinal and prospective designs is required to provide more concrete evidence of the causal mechanisms that link social exclusion and loneliness.

## Supplementary Information

Below is the link to the electronic supplementary material.

Supplementary file1 (PDF 26 KB)

## Data Availability

Data from the European Social Survey are publicly available.
