# Supplementary material for: A social exclusion perspective on loneliness in older adults in the Nordic countries
Source: Eur J Ageing. 2022 Mar 29;19(2):175–88. doi: 10.1007/s10433-022-00692-4 (PMC9156591; doi:10.1007/s10433-022-00692-4)
Supplement: Supplementary file 1 — Supplementary file1 (PDF 26 KB) [file 10433_2022_692_MOESM1_ESM.pdf]

Supplementary material: Supplementary data

Title: A social exclusion perspective on loneliness in older adults in the Nordic countries

Content

Supplementary Table 1: Categories of loneliness per country and survey wave, weighted number of observations

Supplementary table 1. Categories of loneliness per country and survey wave, weighted percentages. Percent (lower confidence interval, upper confidence interval).

Country	Wave (year)	n	None or almost none of the time	Some of the time	Most of the time	All or almost all the time
Denmark	3 (2006)	363	82.6 (78.9, 86.4)	14.0 (10.3, 17.8)	1.4 (-2.3, 5.1)	1.9 (-1.8, 5.7)
Denmark	5 (2010)	382	83.5 (79.8, 87.2)	14.4 (10.7, 18.1)	1.3 (-2.4, 5.0)	0.8 (-2.9, 4.5)
Denmark	6 (2012)	386	84.2 (80.5, 87.9)	11.4 (7.7, 15.1)	2.6 (-1.1, 6.3)	1.8 (-1.9, 5.5)
Denmark	7 (2014)	388	83.8 (80.0, 87.5)	13.9 (10.2, 17.6)	0.8 (-2.9, 4.5)	1.5 (-2.2, 5.3)
Finland	3 (2006)	457	75.3 (71.6, 79.0)	19.5 (15.8, 23.2)	3.3 (-0.4, 7.0)	2.0 (-1.8, 5.7)
Finland	5 (2010)	515	75.7 (72.0, 79.4)	20.4 (16.7, 24.1)	3.3 (-0.4, 7.0)	0.6 (-3.1, 4.3)
Finland	6 (2012)	642	73.8 (70.1, 77.6)	19.6 (15.9, 23.3)	3.4 (-0.3, 7.1)	3.1 (-0.6, 6.8)
Finland	7 (2014)	615	81.8 (78.1, 85.5)	14.5 (10.7, 18.2)	1.5 (-2.3, 5.2)	2.3 (-1.4, 6.0)
Norway	3 (2006)	404	77.5 (73.8, 81.2)	17.6 (13.9, 21.3)	4.2 (0.5, 7.9)	0.7 (-3.0, 4.5)
Norway	5 (2010)	365	82.2 (78.5, 85.9)	14.8 (11.1, 18.5)	1.9 (-1.8, 5.6)	1.1 (-2.6, 4.8)
Norway	6 (2012)	384	83.6 (79.9, 87.3)	15.1 (11.4, 18.8)	0.5 (-3.2, 4.2)	0.8 (-2.9, 4.5)
Norway	7 (2014)	374	81.8 (78.1, 85.5)	15.0 (11.3, 18.7)	2.1 (-1.6, 5.9)	1.1 (-2.7, 4.8)
Sweden	3 (2006)	509	77.2 (73.5, 80.9)	17.1 (13.4, 20.8)	3.3 (-0.4, 7.1)	2.4 (-1.4, 6.1)
Sweden	5 (2010)	414	76.6 (72.8, 80.3)	17.1 (13.4, 20.9)	4.3 (0.6, 8.1)	1.9 (-1.8, 5.7)
Sweden	6 (2012)	532	76.9 (73.2, 80.6)	18.4 (14.7, 22.1)	2.6 (-1.1, 6.4)	2.1 (-1.7, 5.8)
Sweden	7 (2014)	520	75.0 (71.3, 78.7)	19.2 (15.5, 23.0)	3.5 (-0.3, 7.2)	2.3 (-1.4, 6.0)
